# 'Palliative care': a contradiction in terms? A qualitative study of cancer patients with a Turkish or Moroccan background, their relatives and care providers

**DOI:** 10.1186/1472-684X-9-19

**Published:** 2010-09-10

**Authors:** Fuusje M de Graaff, Anneke L Francke, Maria ETC van den Muijsenbergh, Sjaak van der Geest

**Affiliations:** 1 Medical Anthropology & Sociology Unit, University of Amsterdam, Oudezijds Achterburgwal 185, 1012 DK, Amsterdam, the Netherlands; 2 NIVEL (Netherlands Institute for Health Services Research), PB 1568, 3500 BN, Utrecht, the Netherlands; 3 VU University Medical centre (EMGO+), PB 7057, 1007 MB, Amsterdam, the Netherlands; 4 Radboud University Nijmegen Medical Centre, PB 9102, 6500 HC, Nijmegen, the Netherlands; 5 Pharos (Knowledge and advisory centre on refugees, migrants and health) PB 13318, 3507 LH, Utrecht, the Netherlands

## Abstract

**Background:**

Palliative cancer care aims to improve quality of life and ultimately quality of dying, while prolonging life is not an objective anymore when death nears. The question is, however, whether these perspectives on palliative care are congruent with the perspectives of immigrant families with a Turkish or Moroccan background.

**Methods:**

A qualitative design was used as we were looking for the personal views of 'very ill' cancer patients with a Turkish or Moroccan background, their family members and their Dutch care providers. We interviewed 83 people, involved in 33 cases to obtain information about their views, values and norms on 'good care'.

**Results:**

The main concerns about 'good care' expressed by Turkish and Moroccan families were: maximum treatment and curative care until the end of their lives, never having hope taken away, devoted care by their families, avoiding shameful situations, dying with a clear mind and being buried in their own country. Their views conflict, to some extent, with the dominant principles in palliative care, for example, the emphasis on quality of life and advanced care planning, which includes discussing diagnosis and prognosis with the patient.

**Conclusions:**

Patients and their families with a Turkish or Moroccan background often have different ideas about 'good care' than their Dutch care providers. As many of them are aiming at cure until the end of life, they find 'good palliative care' a contradiction in terms.

## Background

The concern for good care for people in the last phase of their lives is common to all eras. But since the 1990 s, health care policy and science have tended to focus more on the essence of palliative care. Ever since 2002, the WHO definition of palliative care has been an approach that improves the quality of life of patients and their families facing the problem associated with life-threatening illness, through the prevention and relief of suffering by means of early identification and impeccable assessment and treatment of pain and other problems, physical, psychosocial and spiritual [1].

The palliative phase is seen by experts as a continuum of care that begins with the diagnosis of a life-threatening condition that can be expected to result in death [2]. Palliative care starts at the beginning of the continuum, but care aimed at prolonging life is often given as well. At the end of the continuum, the patient's needs often become greater and more complex, and the emphasis moves to improving the quality of life. Prolonging life is no longer an objective. Improving the quality of life and, ultimately, the quality of death, also means the effective relief of pain and other symptoms, which often implies the use of opiates. If pain and other symptoms are particularly difficult to treat, the decision is sometimes made to use palliative sedation [3,4].

In order to anticipate the increasing need for care correctly, experts believe that it is important to have a proactive approach right at the start of the palliative care continuum. This is referred to as 'advanced care planning' [5,6]. In order to anticipate the level of care predicted by the care providers, it is important that the patient is not only familiar with the diagnosis, but also informed about the prognosis. Dutch law obliges health care providers to supply the patient with all the requisite information, unless this information is harmful to the patient or if the patient expressly states that he does not want this information. Striving to improve the quality of life includes the choice commonly made in the Netherlands to allow the patient to be cared for at home in the final phase, and to die there [7,8].

All in all, palliative care covers a wide area; it includes pain relief, but also the prevention and relief of other possible symptoms, such as pressure ulcers, breathing difficulties, constipation, anxiety, depression, etc. Palliative care also means that the patient's family will receive psychosocial care to help them to mourn, for example.

The focus on quality of life, open communication and advanced care planning has broad-based acceptance among Western care providers practicing palliative care. The question is, however, whether these perspectives on palliative care are congruent with the opinions, norms and values of non-Western patients. Several studies pointed out that cultural background is important in palliative care, as care providers want to meet individual end-of life wishes which are often culturally determined [9-14].

Relatives of patients with a Turkish or Moroccan background may find it hard discuss the incurable nature of a disease and that family members often do not want the patient to be fully informed [15-21]. In addition, in our first study on this subject [17] we found that terminally ill Turkish and Moroccan patients and their family members often did not understand the diagnosis or prognosis and blamed the GP for not acknowledging the seriousness of their patient's illness. In a second study we found that Dutch GPs and home care nurses often considered communication problems as serious barriers in the access to suitable care for these migrant groups [18]. These previous studies also provided indications that the perspectives on good care and good communication between professionals, patients and relatives often diverged. We therefore decided to conduct a third study, to focus specifically on these divergent perspectives around current cases of patients with incurable cancer. This paper, which focuses on the distinctive views of 'good care' in the palliative phase, presents a part of the results.

The questions that we wish to answer in this article are:

1) What do cancer patients originating from Turkey or Morocco understand by 'good palliative care'?

2). How do Dutch care providers deal with ideas that diverge from the dominant values within palliative care?

## Methods

### Terminology

The description 'people with a Turkish or Moroccan background' includes all residents of the Netherlands who have at least one parent born in Turkey or Morocco. These are the two largest immigrants groups in the Netherlands. Although Turkish and Moroccan cultures diverge, in this study both groups have been studied together, as the first generation in both cases came to the Netherlands between 1965 and 1985 as immigrant workers and have undergone a comparable process of socialisation. Their socioeconomic positions in the Netherlands are also comparable, and both groups come from a Muslim culture [22].

The term care providers includes general practitioners (GPs), medical specialists, nurses and social workers who are directly involved in the palliative care of cancer patients and their families. By the dominant principles within palliative care, we mean, for instance, the emphasis on quality of life and the importance assigned to advanced care planning within the 'palliative care continuum'.

### Research methods

A qualitative research design was used, because we were looking for insights into personal ideas and experiences of people on the subject of the study, and also because the number of cancer patients with a Turkish or Moroccan background in the Netherlands is still limited. In order to answer the questions, we held semi-structured interviews with patients, their families, and those care providers directly involved. We were aiming at retrieving the emic views [23], which means, in this case, the way in which the care provided was perceived on the one hand by the Turkish and Moroccan families within their own cultural and social systems and, on the other hand, by the care providers within the Dutch professional culture.

### Ethical aspects

The research has been formally approved by the Medical Ethical Committee of the most involved region (METC Zuidwest Holland, nr 07-113, 2008) and by the ethical committees of the involved hospitals.

The patients' and family members' consent to the interviews was recorded on tape, at the beginning of the interview session. We decided on this form of consent, because it emerged from earlier research that immigrant people with a low level of literacy preferred not to sign a declaration that they were unable to read for themselves [24].

### Recruitment and research population

Patients and their families were mainly contacted indirectly through GPs, and professionals working in hospitals or homecare organisations, as the question whether the person involved was suffering from cancer could not easily be put to them directly. The first contact between the first author and care providers often took place at regional network meetings for palliative care or at national symposia. In addition, we composed a short letter of introduction, which was sent by e-mail and post to care providers who might be available for interview. A shortened form of the introductory letter was translated into Turkish and Moroccan Arabic for the patients. They received this information through their care providers and, in some cases, directly from the researcher.

Interviews were held on 33 cases of patients with incurable cancer, 27 of these cases concerned patients of the "first generation immigrants", and six other cases concerned patients of the "second generation". In some cases interviews were held with two or three interviewees together.

The age of the patients varied. Fourteen patients were younger than 50 years, thirteen were 50-70 years old and six patients were older than 70. Seventeen patients died at home, seven in hospital, two in a hospice, three in Turkey and four were still alive when we stopped the phase of data collection.

In 6 of the 33 cases, we were also able to speak to the patient in person. In the other cases, this was not possible, because the care provider involved or the GP did not want the patient to be bothered by the interview.

In addition, five male and 25 female family members and 47 care providers were interviewed. The care providers included 17 GPs, 19 nurses, five specialists, four social workers and two clergymen. More information about the background characteristics of the cases can be found in Table 1.

**Table 1:** Characteristics of the cases

Case number	Background	Illness	Patient Interviewed	Family interviewed	Professional Interviewed
1	Tu*	mesothelioma	------	daughter	GP

2	Mo**	bladder cancer	------	------	GP

3	Tu	stomach cancer	------	------	GP

4	Mo	bronchial cancer	patient	wife brother-in-law	pain specialist nurse home care nurse GP

5	Tu	breast cancer	patient		social worker.

6	Mo	lung cancer	------	dife	GP

7	Tu	lung cancer	------	-------	pastoral worker

8	Mo	stomach cancer	------	wife	GP social worker

9	Tu	brain tumor	------	-------	nurse GP

10	Tu	lung cancer	------	-------	oncology nurse

11	Mo	breast cancer		-------	oncology nurse

12	Tu	stomach cancer	------	-------	hospital nurse

13	Tu	breast cancer	patient		social worker

14	Mo	brain tumor	patient	mother 2 sisters brother	GP oncologist pastoral worker

15	Mo	lung cancer	------	daughter	GP

16	Mo	breast cancer	------	daughter	GP

17	Tu	bowel cancer	------	wife son	oncology nurse

18	Mo	stomach cancer	------	sister	home care nurse

19	Mo	bowel cancer	------	daughter	

20	Tu	stomach cancer	patient	daughter	hospital nurse

21	Mo	lung cancer	------	daughter	social worker oncologist pain specialist transfer nurse

22	Tu	bone cancer	------		GP home care nurse

23	Tu	lung cancer	------		home care nurse

24	Tu	lung cancer	------	wife son daughter	GP

25	Mo	bowel cancer	------	wife	GP

26	Tu	lung cancer	------	wife daughter	GP

27	Mo	stomach cancer	------	daughter	GP home care nurse

28	Mo	brain tumor	------	daughter	GP

29	Mo	bowel cancer	------	husband sister-in-law	medical specialist

30	Tu	bowel cancer	------		oncology nurse specialist

31	Mo	ovarian cancer	patient	daughter	oncology nurse home care nurse GP wound nurse

32	Mo	breast cancer	------	sister	

33	Mo	breast cancer	------	daughter	nurse home care nurse

*: Tu = Turkish patient **: Mo = Moroccan patient.

### Interviews

For the semi-structured interviews we developed a topic list containing questions on a few facts (e.g. disease characteristics, treatment and care trajectory, family and professional context) and experiences in the process of decision making. So, we asked the respondents to describe decisions and actions during the treatment and care trajectory and to reflect on these decisions in the light of their personal views and values. Finally, we asked them what emotions these reflections aroused and what concerns lay behind these emotions, as these often reflect the respondents' vital views, values and norms.

The length of the interviews varied from less than a half hour (in the case of busy care providers) to two hours (with patients and their families who wanted to tell their stories in detail). The first author interviewed care providers at their work and the patients and their families at home or in hospital. In most cases the interviews were held in the Dutch language. However, a professional interpreter was recurred to four times, and family members acted as interpreters four times. The interviewing was done in 2008. We stopped recruiting new interviewees after we had obtained theoretical saturation [25] on the main theme of this article, i.e., the perceptions on 'good care'.

### Analyses

As usual in qualitative research, data analysis already started after the initial interviews, as part of a cyclic process of "data collection - analysis - further data collection and analysis". The Dutch-language parts of the interviews were all typed out. The first author analysed all interviews, while the second author analysed the interviews of the first thirteen cases and a third of the remaining twenty cases. Both authors independently described their individual analyses in "memos" and discussed any apparent disparities until agreement was reached. Besides, the first author coded the data of all the interviews systematically with the help of MaxQda [26]. This software programme easily sorts relevant fragments and links these to other fragments with the same keywords or codes within or between interviews. In the coding process several keywords were used expressing interviewees' views on the care delivered, the communication or decision making, such as: curative care, hope, trust, shame, religion, keeping a clear mind, relation with the home country, failure to communicate etcetera. Some fragments of interviews were assigned several keywords.

Halfway through the research, the first author described the main outcomes of the analysis in an interim report. This report was discussed with the four authors and with the members of the advisory committee (a GP, an oncologist, one Turkish and three Dutch advisors with expertise in this subject, two researchers and two policymakers involved in this field). Such "peer debriefing" [27], is important to improve the quality of the analysis and to reduce one-sided interpretation of the data.

In addition, we discussed the findings and interpretations with representatives of the Turkish and Moroccan communities in the Netherlands in fifteen discussion meetings. This activity also turned out to be useful to improve the quality of the analyses and to verify what had been found.

## Results

### Views of patients and their families on 'good care'

The views on 'good care' of the seriously ill patients and their relatives with a Turkish or Moroccan background diverge from those of the Dutch care professionals on several points.

#### Curative care up to the end

The interviews showed that, for the patients and their families, 'good care' implied that care providers should use their knowledge and skills until the very end to try to cure the patient or, at least, to keep him alive as long as possible.

*He was really too weak for a third and fourth time, but we said, we'll just go on, we believe in it; he'll get better, we won't stop, we'll go on (daughter of a Turkish male patient)*.

Whether patients and their families actually believe that they will receive 'good care' is affected by previous care experiences. Respondents who have rather low expectations often perceived medical shortcomings that - in their opinion - brought about their present situation. Doubts about the expertise and the commitment of Dutch doctors are often exacerbated by the stories of other immigrants who compare Dutch health care with the opportunities in their countries of origin or other European countries.

*He went to a big professor in Istanbul. And then they said, why did you let them take away a piece of your lung? That makes it worse. If you hadn't done that, we could have tried different treatment (son of a Turkish male patient)*.

#### Maximum care

Patients and their families do not just want curative care; 'good care' implies maximum medical treatment and diagnosis. This often means that people want a 'second opinion' and will ask for medical tests or (chemo) therapy at a very late stage.

*We asked for a second opinion and we wanted the chemo cure, just to see whether it would work. Until we said, yes, it's no good. Of course, you have to accept that. He couldn't say it, we did that for him. It was a battle, over and over again, we are still going on. We won't accept 'no'. Hoping that it might work, that some other treatment might be possible (sister of a Moroccan male patient)*.

Wanting the best possible treatment and diagnosis goes together with the idea that you must fight to the last. Respondents stated that they saw that as a religious commandment. The duty of the patient to fight for his life obliges the family to do everything possible to save their relative. They want the patient to eat and drink and, if possible, to stay mobile.

#### Keeping hope alive

Another view of 'good care' is that care providers should not take away the hope of recovery by talking directly and openly about the negative prognosis. If hope is removed, then the family is afraid that the patient will 'give up'.

*You can say it, but then tell us (relatives), as, if you tell him, he'll give up (wife of Turkish male patient)*.

Family members want to keep hope alive in the terminally ill patient, because hope can give him strength to get through this very difficult period.

*When I heard that the tumour was malignant, I couldn't tell him and I asked my doctor not to discuss this with my father, he needs morale, hope (daughter of Turkish male patient)*.

Some respondents also say that they cannot take away the patient's hope for religious reasons. According to them, it is for Allah to decide when someone is going to die; life and the possibility of recovery are in Allah's hands. This is why families ask care providers to be cautious in giving information. Maintaining hope is more important than a complete picture of diagnosis. However, often some of the family are informed.

*I didn't tell my mother either. I had all the information, I knew what was happening. I did tell my father what the possibilities were. But I don't believe my father ever told her (daughter of Moroccan female patient)*.

The desire not to speak about the diagnosis and prognosis or only in very veiled terms can sometimes have consequences when a patient is discharged from hospital. Relatives want to prevent him hearing that he is allowed home 'because there is nothing else the doctors can do'. Within the family, they are often just as cautious about giving information. Certain relatives (for example, those responsible for interpreting and bodily care) will be informed, others will not.

#### Personal attention and being treated with respect

'Good care' also implies personal involvement. A good care provider will be receptive to contact with the patient and his family, ask about their experiences, listen to their questions and answer them. In addition, a good care provider takes time for them and waits patiently if things have to be translated.

*He was a very good doctor, one of the old school, more experience, you could see that straight away, more patience. A doctor should give you the feeling 'We are here for you'. Other doctors were more like butchers, they were in a hurry (daughter-in-law of Moroccan male patient)*.

*There were two nurses, they had no feel for social skill, they were, how can I put it, they were a bit clumsy, it was a conveyor belt, as the saying goes, but with them you could feel emotions, warmth and love and she's still got those nurses' (daughter of Moroccan female patient)*.

The importance that people attach to personal attention and respect is not in itself unique to patients with a Turkish or Moroccan background. However, it does seem to be characteristic of some patients from these target groups that they have the idea that Dutch natives get more attention and respect in the Netherlands than do immigrants. They get this idea, for example, from the order in which people in waiting rooms are called in (When is it our turn?), from the way beds are assigned (We wanted a single room, too) to the hospital discharge (I can't stay in hospital any longer because of my insurance). These ideas are often based on stories from fellow patients.

*My question is why they take such care in Germany (a country where many Turkish people also live, FMdG) and not in the Netherlands. Yes, maybe it's just us, maybe it's different for the Dutch and only like this for Surinamese, Moroccans and Turks (son of a Turkish male patient)*.

#### Devoted care by the family

Good care implies that you can call on your family. Your family is obliged to care for their sick relative properly. It is mainly women who take the caring on themselves. Male patients are sometimes washed by their brothers and sons, but more often by their wives and daughters.

*And my brothers? They weren't really in the picture, they've got their own stuff, it was more my sisters and I who were involved in the caring (sister of Moroccan female patient)*.

Sometimes the female relatives take turns, but, more often, one of them takes on the lion's share of the caring. Although it is often assumed that in Turkish or Moroccan families women perform the care in the home while the men maintain contacts with the outside world [28], this rule does not always apply. In the cases in this study, it was mainly the women who were in contact with the GP and the hospital.

In most families, a pattern of caring relationships predates the patient's illness. This then serves as a basis, though hospitalisation, the intervention of the GP and sometimes home care can change this pattern.

Personal physical care and household tasks are seldom handed over to other people within the Turkish or Moroccan communities. It is, however, their duty to provide social care. Relatives visit often, coming to pay attention to 'the invalid' and to divert him.

*My mother had a lot of visitors. The doctors were frustrated with us. They said. 'There are such a lot of relatives dropping in, your father needs rest'. Yes, well, the younger ones understand that. They came by, just to pay their respects and left again. But the older ones, for example, my uncles and aunts, they came and sat by the bed. And my mother enjoyed that too (daughter of Moroccan male patient)*.

Sometimes, relatives come from other parts of the Netherlands or from abroad and stay with the family for a few weeks. Patients who used to be active in the mosque also get visits from members of the congregation. But people from the mosque do not get involved in the caring, either. The rule is - a devoted family cares for the 'seriously ill' patient.

#### Avoiding shameful situations

'Good care' implies that, as far as possible, shameful situations are avoided. Many families find it difficult to hand over care in the home to professionals as they are afraid that there will be gossip in the local community.

*They didn't want a district nurse because they were afraid of gossip, gossip, gossip. When it was made very clear to them that these were people who didn't come from our town, the problem suddenly became a lot less threatening. The local home care service hired a Moroccan woman from elsewhere, who spoke Berber and Arabic (GP of Moroccan male patient)*.

The family dislikes the idea of using home care, not just because they would feel that they had failed, but also because they would see it as 'shameful' to 'expose the patient to strangers'. Particularly when the care professional is of the other sex.

*If it is an older woman, then they want a woman to come. Because, however sick you are, you are not allowed to have a man at your bedside. And vice versa (sister of a young female Moroccan patient)*.

#### Dying with a clear mind at the time appointed by God

Another prevailing opinion on 'good care' is that pain relief or sedation must not go so far that that the patient becomes dull-witted or unconscious. Various respondents have said that the patient on the point of death must be clear-headed enough to take leave of his loved ones and to forgive them. They also think it important that the patient can appear before Allah in a clear state of mind in order to answer for himself. This is why people often have problems with opiates being given and deep sedation is often refused.

*From the moment that her brain was affected, they discussed with us whether we would not rather keep her asleep. I had the feeling that we were being put under pressure, that we couldn't really make our own choices. I didn't want that. It is not permitted to let someone meet death like that (daughter of Moroccan female patient)*.

A cautious approach to the use of sedatives or opiates can also go together with the fear that the Dutch may be giving drugs to speed up the process of dying. It is not only that people have problems with medical treatment that might (possibly) curtail life, even stopping treatment to prolong life is seen as being in conflict with the religious commandment not to take life.

*I can imagine that if I were in a stage where I just didn't want to go on, then I could just stop taking the medicine. But for my parents, that's not an option. This is very different from Dutch culture and it was new to me, too. It is not allowed, you are not allowed to commit suicide in Islam, you have to do everything to, as long as you're still alive, it's good, you are not allowed to end a life (daughter of a Moroccan female patient)*.

#### Care in the country of origin

Many patients and their families appreciate the health care in the Netherlands, not least because they are insured for the expense that illness brings in its wake. But many families also attach importance to contacts with care providers in their countries of origin. Some of them, as mentioned above, want a second opinion in their own country. Others hope that a holiday in their own country will help, that the sun, mountains, family and old friends will do the patient good, physically and emotionally.

Some families believe that, in their own countries, their contacts will allow them to get a better grip on health care. This is particularly true of families with money and connections.

*My father's oldest brother came up with the idea. He said, I know hospitals, because he studied in Turkey, he's got friends who are doctors in Turkey. And those doctors have friends who are special for those diseases. They come from America and so on. My uncle says to my father, get up, go there, you've still got a chance (son of a Turkish male patient)*.

Another contributory factor is that in the patients' countries of origin, they can stay longer in hospital, thus doing their best to get maximum treatment. Also, some patients choose to die in their own country. Preferably in the presence of both the family living in the Netherlands and those who have remained in their country of origin.

Sometimes the decision to return for care to their own country is taken too late; the journey is too exhausting and/or airlines will not take the patient at that stage.

#### Burial in the country of origin

For many families with a Turkish or Moroccan background, 'good care' implies, in the last instance, burial in their own village or town in their country of origin. Various respondents have indicated that they have taken out insurance for this with special Turkish or Moroccan organisations. These organisations will, if required, arrange the entire funeral. Some Dutch undertakers employ specialists who organise burials in Morocco and Turkey.

*We had an insurance. You just call their number when you need them and everything is organised. Then I said goodbye to him and he was taken to another room. The man came the next morning, he was ritually washed and laid out in the mosque and the next day I was able to go with him to Morocco (wife of a male Moroccan patient)*.

Good final care involves ritual washing and wrapping in cloths by Muslims of the same sex; after this, the funeral in the country of origin is always arranged by men. Therefore, little is expected here of the Dutch care providers, but speed may be necessary in taking the deceased as quickly as possible to his last resting place.

*In our culture and according to our faith, once someone dies, they must immediately be undressed and wrapped in cloths and buried as soon as possible (daughter of a male Turkish patient)*.

Travel to the country of origin and burial there are an expensive business for which families sometimes get themselves into debt.

### Reactions from care providers to these specific views

How do the Dutch care professionals react to these views? From the interviews with 48 care providers who were closely involved in the palliative care of the 33 patients, it appears that many of them realise that there is often a question of 'different ideas'.

#### Curative care until death

The care providers in the cases we studied are aware that patients from a Turkish or Moroccan background often wish to continue for a very long time with curative care aimed at prolonging life.

*That was what the children wanted to know, too. Could they be sure that father would receive the best possible care? People don't want to go to the hospital, but they don't want to miss out on any possible chances. It's no good saying that there is nothing more that can be done, you must do everything possible and, then, if father does die, everybody is satisfied (GP of Turkish male patient)*.

In practice, the desire for curative care to prolong life regularly stands in the way of any joint investigation and decision making on the subject of the various kinds of palliative care.

*I am dissatisfied as I had hoped that I could arrange for discharge from hospital to everyone's satisfaction. Then it's not nice to see that it hasn't worked. That people were so upset at home. I think that's sad. But what else could I have done? I don't know. There were moments when I thought, 'Am I being used... umm... or are we working together?' And I never did get it under control (transfer nurse of a Moroccan male patient)*.

The preference for curative care can sometimes result in patients in the final stages still ending up in hospital. GPs and other care providers involved often find this a problem, especially when the communication between inpatient and outpatient health care breaks down. In their eyes, this negatively affects the quality of care.

#### Maximum treatment

Care providers often mention that these patients and their families are looking for maximum medical treatment. They deduce this from the efforts that the patient makes to stay alive, and from the patients' and relatives' reactions to advices from doctors and nurses. Care providers find it hard to deal with, if patients or their families ask for treatment which the professionals regard as pointless.

*I know that it was very difficult for me to convince them of the fact that radiotherapy was really not an option, that it was no longer possible. They took the attitude, more or less, 'it worked in the past, so it should work again' and 'can't we go to another hospital, then?' (GP of a Moroccan male patient)*.

#### Keeping hope alive

The care providers we interviewed have generally noticed that the family do not want to take any remaining hope away from the patient. They also come across situations where the patient or the family do not want third parties (e.g. relatives not directly involved in the caring or people outside the family) to be told about the negative prognosis. The reactions of the care providers diverge. Some doctors accept the request for silence because they realise that not everybody can deal with the whole truth and hope can be beneficial for the patient. Some of the doctors and other care providers accept the family's wish as they assume that the family knows the patient best or because they are dependent on translations by family members. Others find it more difficult, and see it as 'denial' or 'out of date'.

*There was at that moment no possible opening for a real discussion of what the prognosis was. They were all deep in denial, really old-fashioned, like we had with Dutch patients too, thirty years ago (GP of Moroccan female patient)*.

Some doctors in attendance do not want to take the wishes of the family into account, because, in their opinion, it is better for all patients if they are fully informed. Only then can they be involved in decision making on the treatment to be carried out.

*I think that a patient must know what the matter with him is. And nobody should talk about a patient without the patient being aware; this leads to what in your terms is a conspiracy of silence (oncology specialist of Turkish male patient)*.

Nurses and social workers often seem to have less difficulty with this request for silence than do many doctors. Their discussions are often about subjects other than medical treatment, and, partly for this reason, they seem to be better able to understand the underlying reasons.

*I know that many Turkish and Moroccan patients, people, do not want to talk about the subject of 'dying'. But look, I talk with them about everyday things, things to do with care, yes, general things (nurse of Turkish patient)*.

Some of them, however, find that it is difficult to maintain silence if their relationship with the patient becomes confidential and he then asks for information.

#### Attention and respect

Some care providers from the cases we studied recognised that discussions had not always been conducted with respect. The trust that is essential to building up a good care relationship is missing at a moment like this.

*I don't know what made them mistrustful, but I think that they thought, 'We are being treated as though we're inferior (nurse of Turkish male patient)*.

But they also believe that personal attention and treating people well is part of 'good palliative care'. Opinions on of what treating people well means, however, can differ. While families believe that they deserve as much respect and attention as the patient many care providers believe that they are there primarily for the patient, because it is the patient who should, as far as possible, keep control of the care.

*A patient is for me the central point. And I often start by saying, I will only talk to you. If other people call and say, explain what's going on, then I will refer them to you. If you find it difficult to explain things to your family and friends, then I will be happy to help you, but I am not going to explain it to them myself. Because I want the patient to keep control of his part of the treatment, I want him to have the same information as his family (oncologist of Turkish male patient)*.

They sometimes get irritated by family members, especially if the relatives present themselves as spokesmen or get in the way of direct contact with the patient. Moments like this reveal that Dutch care providers interpret 'respect' differently from the families.

*As I came in, I was lectured by her in the hall on what I could or couldn't discuss with him; it was as if she were giving me instructions (GP of Moroccan male patient)*.

#### Devoted care by the family

The health care providers in the cases we studied appreciate the fact that the families want to care for the patient, although some of them remarked that the caring was mainly the responsibility of the women.

And there were sons as well, but they didn't do that much. Well, sons in general tend to do less. Certainly Moroccan sons, I'm afraid (oncology specialist of Moroccan male patient)

More of a problem for the health care providers was that the relatives' duty to care for the patient could become too much for some of them in the long term, but that this would be impossible for them to discuss openly. In families where caring was done as a 'matter of course', health care providers can hardly pre-empt the problems that they foresee but which have not yet occurred.

*It's more of a case of reacting when a problem occurs, unfortunately you can't really take preventive action, it's more dealing with the problems as you meet them (GP of Moroccan female patient)*.

Some health care providers remark that their own structured working methods do not fit in with the families' ways of working or perceiving the situation.

*I keep trying to see whether it's possible to talk about the possibility that things might be coming to an end. After all, it's typically Dutch to want to arrange things and, in some sense, to say goodbye ahead of time. The Dutch are quicker to do that, often they suggest something themselves, like shouldn't we put a bed downstairs. And then I order it. With this family you have to be much more careful with everything, they need time to think it over (nurse of a Moroccan male patient)*.

In addition, care providers say that it can be difficult to come to any arrangements in families with a Turkish or Moroccan background. For example, some people have a lot of relatives at home, others don't. Some people don't always open the door or answer the phone, and sometimes things are mistranslated. However, sometimes it goes really well and there's a close working relationship between various care providers and the relative who is the main point of contact.

*We appointed one person to be the main point of contact, her sister. She's a bright girl, I know her well as she suffers from stomach problems and so I've seen her a lot. She was going to help as her mother still hardly speaks any Dutch (GP of Moroccan young female patient)*.

#### Avoiding shameful situations

Care providers realise that shame is an important issue for many patients and their families; shameful situations need to be avoided. Some care providers, however, find certain social rules irritating, like not taking a proffered hand or the women leaving room when male visitors arrive. Doctors notice that the fear of shameful situations can put constraints on professional care and certain types of medical examination.

*I noticed that I was carrying out fewer physical examinations than I would with someone with a Western background, say. Actually, I don't think that's right, so now I do what I normally would, I want to look at her stomach, I have to sense what she finds acceptable or not, and what I think is medically necessary and then find a happy medium (GP of Moroccan female patient)*.

Nurses also experience constraints on care, but try, nonetheless, to take feelings of shame into consideration. Often, their bond with the patient and the family grows stronger as time goes by and they increasingly become a trusted figure both for the patient and the family. District nurses sometimes come initially just for specific technical advice and then slowly grow into a situation where they are also allowed to give physical care as well.

*So, at first*, *he looked after his own personal care. But then, after a few weeks, this got more difficult, and he managed to admit it. But we weren't allowed to do it for him yet, Then, after another few weeks went by before he admitted that he really couldn't do it any more, then we were allowed to do it, the three of us, with him keeping his underpants on under the shower (nurse of a young Moroccan male patient)*.

#### Dying with a clear mind without hastening death

Many care providers in the cases we studied are familiar with the wish that the patient should appear before Allah with a clear mind and therefore would rather not be dull-witted at the moment of death. But sometimes doctors are faced with a moral dilemma if the patient is visibly suffering but the family rejects drugs. Is good care less important than a good start in the hereafter? Sometimes doctors believe that their responsibility is to provide good care in the here and now.

*I said, if I don't do this than I'm committing a criminal offence, as I am obliged to do my best as a doctor to alleviate his suffering. If you carry out euthanasia without permission, you are acting against the law, this isn't euthanasia and if I don't help him properly now with the drugs that will make him sleep, then I am not a good doctor and then I am committing a criminal offence. Then they accepted it (GP of a Moroccan male patient)*.

In other situations, people look for solutions somewhere in between. For example, when the daughters of a Moroccan female patient who was seriously delirious said that their faith did not permit deep palliative sedation. The strategy then was to administer a heavier dose of sedatives at night to give the woman some rest, and less during the day so that contact between mother and daughters was still possible.

*A needle was put under her skin with a pump containing a very high dosage of drugs to make her sleep. But that was adjusted during the day so that she could still interact with her daughters. The daughters did not want her to lose consciousness, so she wasn't completely in a coma. She died shortly afterwards (GP of Moroccan female patient)*.

Many care providers also know that these patients prefer to avoid opiates because they are afraid that they will result in cutting short the life of the patient. Some of the care providers have experienced situations where the families are afraid that doctors will carry out euthanasia on their own initiative. Extra time has to be spent in discussion and giving information to convince the family that nobody has any intention of shortening the life of their loved one, but that relief of suffering is the aim.

*Then I explained that we were not allowed to give anyone a lethal injection just like that, only that you are obliged to, if someone can't breath or is in a lot of pain and God or Allah says that too, because you are not allowed to let anyone suffer unnecessarily. It is my duty to see that no-one suffers unnecessarily. They accepted that (GP of Moroccan male patient)*.

#### Care in the country of origin

Care providers working with these target groups often find that patients look for help in the meantime in their own country.

*My experience is that many Turks want very much to go back to Turkey to get a second opinion there and I don't think that's a bad thing at all. I can imagine very well how they feel (GP of Turkish male patient)*.

Generally, care providers understand this choice and help to bring it about. But they do find the advice and intervention of their Turkish colleagues sometimes confusing as well, as they often recommend a more curative policy. Sometimes care providers can see that a trip like this is going to disrupt the treatment in the Netherlands. If they believe that the plan is irresponsible, then they try to persuade the family that they should stay in the Netherlands.

*Then the daughters said that he should go to Morocco, because he would be treated there and he could die there too. I said, 'You decide, but I don't know what the medical and palliative care is like there'. I gave a brief outline of what I as their GP could do, together with the hospital, with pain relief and in the case of mental confusion. And that I was worried about what it would be like in Morocco. Then, after a while, they decided to stay here after all (GP of Moroccan male patient)*.

Often the extra attention and information result in the family making a joint decision to stay in the Netherlands or to postpone the trip. But it can also happen that any discussion of their choice appears impossible, as their desire to go back to their own country is so overwhelming that any objections on the part of the care provider are simply not heard.

*I didn't get a 'fit-to-fly' recommendation, and then the ambulance won't take him to Schiphol (airport) because the airline probably won't take him. The family was very angry; they had already bought a ticket for him. Then we decided to take the man off our books. We removed the oxygen. That was OK. Then we started using morphine plasters instead of the morphine pump. I found out for them which ambulance taxi they could call. The sister had told met that he was taken into hospital in Turkey and put on a drip and that the doctors said he would get better, but he died anyway (cancer nurse of Turkish male patient)*.

If the aim of the journey is to take leave of important family members, but the journey is impossible because of the patient's poor health, then care providers are sometimes prepared to help with bringing over Turkish or Moroccan relatives to the Netherlands. This could be by writing a recommendation for a visa, for example, or actively interceding with various funds to get the expenses of the trip paid for.

#### Burial in the country of origin

The care providers involved generally know that those of their patients with a Turkish or Moroccan background must be laid out after death according to Muslim rituals and that they generally want to be buried in Turkey or Morocco. They are sometimes surprised that the responsibility for the body of the deceased is so soon handed over to the contact person from the mosque.

*My experience with patients is that have already arranged all this. Before you know it, the imam is there to take over (GP of Moroccan male patient)*.

As some of the family are travelling to their country of origin for the funeral, there is little opportunity for regular aftercare. Some care providers see this as a missed opportunity, others have got used to it.

*With Dutch patients, I would go to offer my condolences, but they had left for Morocco pretty quickly. I put a note in their letterbox to ask if they would get in touch with me. They appreciated that, but I felt it took a long time. Then I thought, maybe I should call myself? I don't want to intrude. That was the final phase for me (GP of Moroccan male patient)*.

## Discussion

Important elements of 'good care' in the palliative phase for people with a Turkish or Moroccan background are generally: curative treatment till the last moment, maximum care, keeping hope alive, attention and respectful treatment, avoiding shameful situations, dying with a clear mind without treatment that might shorten life, care and burial in the country of origin.

Dutch care providers often see the desire for curative treatment until death, the wish for maximum care and hope of recovery till the end as obstacles to joint decision making on palliative care. Care providers sometimes feel that the communication is handicapped by a relative acting as interpreter and person in charge. Some have difficulties with the fact that families attach more importance to avoiding shameful situations than to assuring quality of care. Care providers sometimes have differences of opinion with relatives, as their views on dying with a clear mind and refraining from life-shortening procedures do not always correspond with their own professional values concerning the relief of suffering. Besides, care providers notice that discussing these subjects can be complicated by the image that patients and families have of the Netherlands as a country where euthanasia is practised.

Generally speaking, care providers are receptive to adjusting palliative care administering to the wishes of their patients, but sometimes the care providers' values might hinder them in accepting views rooted in opposite values. This study showed that contradictive views on good care are connected with cultural values of aims and means of care. The main contradictive values are presented in a Table 2

**Table 2:** Contradictory values for aims and means at the end-of-life for Dutch professionals and families with a Turkish or Moroccan background.

Values of Dutch professionals	Values of families with a Turkish or Moroccan background
Improving quality of life	Striving for cure up to the end

Fully informing the patient to reach shared decision making and to realize advanced care planning	Keeping patients' hope alive, therefore the family decides how much information can be given to a patient

Giving sufficient pain and symptom relief	Ensuring that the patient dies with a clear mind

Giving optimal care in the Netherlands	Using opportunities for care in the Netherlands as well as in the country of origin

In case a patient is not aware of the diagnosis, this may be in accordance with his or her personal values and wishes. However, a serious disadvantage may lie in the fact that unawareness of approaching death can result in a non-optimal use of all the possibilities of palliative care [29]

How far are these results specific to the groups studied and the Dutch context? Recent studies on the palliative care needs of specific populations in Australia indicate that open communication and care planning with the patient are more likely to meet the needs of English speaking people than those with another background [30-37]. While Australian researchers have emphasized the language problems [38], Americans often explain cultural differences in terms of ethnicity [39], Asian studies stress the voice of the families thus overlooking the autonomy of the patient [40,41], and Europeans explore the influence of religion [42,43]. Indeed, many Turkish and Moroccan patients and family members in our study indicated that their views were related to Islam, while their Dutch care providers thought that faith, in the broadest sense, was responsible for not accepting the 'modern' vision of communication and care in the final phase of life.

The assumption that views on palliative care are influenced by religious background is confirmed by studies showing that African immigrants in the US and England refer to their Christian beliefs if they resist advanced care planning and an open discussion on diagnosis and prognosis. They prefer extending life with all possible measures and rely on the family as surrogate decision makers [44-47]. However, studies comparing religious doctrines and directives on end-of life decisions for Christians, Muslims and other believers reveal that instructions from holy books and religious legislative bodies on issues like curative care up to the end-of-life and dying with a clear mind, still allow for a variety of interpretations [48].

The open and direct manner of communication of care providers in this study is also related to the Dutch system of health care, which provides everyone with a GP [49]. The care providers are used to the liberal Dutch society where self-determination and consensus are highly valued [50-52]. Dutch care providers are, in general, proud of these 'achievements'.

However, perceptions on end-of-life decisions and actual medical practices vary across multicultural Europe [53,54,43]. We would recommend that care providers place their own perceptions and practices in perspective, and consider the religious and cultural views of their patients and family members [49,55]. Care providers have to keep in mind that their own views on open communication of an infaust diagnosis and prognosis may not be the norm for everybody.

A limitation of our study is that professionals, patients and relatives who are dissatisfied with the care provided and mutual communication were probably less inclined to participate in our study and therefore are underrepresented. But we suspect that people in these target groups who are less motivated or less satisfied would have had similar or even worse communication and decision making problems than our respondents. We believe our presented findings are applicable to other Turkish and Moroccan immigrants and their care providers in the Netherlands.

## Conclusions

This study showed that although Dutch care providers and the families of cancer patients with a Turkish or Moroccan background all wish to provide good care to their patients, they might disagree on the aims and preferred means of 'good care'. They might dispute on the aims of treatment - cure or quality of life -, on what information will be given to the patient, or on what treatment is indicated when death is approaching.

Of course our analysis of different views and values of Dutch care providers versus Moroccan and Turkish families on care at the end of life should be handled with care: every patient and family is unique and care providers also diverge from each other. But Western-oriented care providers should be aware that dominant principles in palliative care such as emphasis on quality of life and advanced care planning are not blindly adopted by patients from a non-western origin. In order to deliver good care professionals should be aware of their own culture-related values and norms and curious to get to know the views of 'others'. Taking time and creating opportunities to question mutual expectations, wishes and fears can help to avoid frictions and lead to strategies and care interventions acceptable to all parties involved. Care providers should accept that for some people 'good palliative care' can be a contradiction in terms, as 'good care' for them must be directed to recovery.

## Competing interests

The authors declare that they have no competing interests.

## Authors' contributions

FMdG designed and conducted the study, performed and analyzed the interviews and wrote the manuscript. ALF contributed to the design of the study, interpretation of the data and the critical revision of the manuscript.

METCvdM and SvdG commented extensively on the design of the study and on the drafts of the manuscript. All authors read and approved the final manuscript.

## Pre-publication history

The pre-publication history for this paper can be accessed here:

http://www.biomedcentral.com/1472-684X/9/19/prepub
